# Genotype-phenotype correlations among *BRCA1 *4153delA and 5382insC mutation carriers from Latvia

**DOI:** 10.1186/1471-2350-12-147

**Published:** 2011-10-27

**Authors:** Grigorijs Plakhins, Arvids Irmejs, Andris Gardovskis, Signe Subatniece, Santa Rozite, Marianna Bitina, Guntars Keire, Gunta Purkalne, Uldis Teibe, Genadijs Trofimovics, Edvins Miklasevics, Janis Gardovskis

**Affiliations:** 1 Hereditary Cancer Institute, Riga Stradins University, Dzirciema Street 16, Riga, LV-1007, Latvia; 2 Oncology Clinic, Pauls Stradins Clinical University Hospital, Pilsonu Street 13, Riga, LV-1002, Latvia; 3 Surgery Clinic, Pauls Stradins Clinical University Hospital, Pilsonu Street 13, Riga, LV-1002, Latvia; 4 The Centre of Health Economics, Ministry of Health of the Republic of Latvia, Duntes Street 12/22, Riga, LV-1005, Latvia; 5 Department of Oncology, Daugavpils Regional Hospital, Vasarnicu Street 20, Daugavpils, LV-5417, Latvia; 6 Oncology Clinic, Liepaja Piejuras Hospital, Jurmalas Street 2, Liepaja, LV-3401, Latvia; 7 Department of Physics, Riga Stradins University, Dzirciema Street 16, Riga, LV-1007, Latvia

## Abstract

**Background:**

Mutations in the high penetrance breast and ovarian cancer susceptibility gene *BRCA1 *account for a significant percentage of hereditary breast and ovarian cancer cases. Genotype-phenotype correlations of *BRCA1 *mutations located in different parts of the *BRCA1 *gene have been described previously; however, phenotypic differences of specific *BRCA1 *mutations have not yet been fully investigated. In our study, based on the analysis of a population-based series of unselected breast and ovarian cancer cases in Latvia, we show some aspects of the genotype-phenotype correlation among the *BRCA1 *c.4034delA (4153delA) and c.5266dupC (5382insC) founder mutation carriers.

**Methods:**

We investigated the prevalence of the *BRCA1 *founder mutations c.4034delA and c.5266dupC in a population-based series of unselected breast (n = 2546) and ovarian (n = 795) cancer cases. Among the *BRCA1 *mutation carriers identified in this analysis we compared the overall survival, age at diagnosis and family histories of breast and ovarian cancers.

**Results:**

We have found that the prevalence of breast and ovarian cancer cases (breast: ovarian cancer ratio) differs significantly among the carriers of the c.5266dupC and c.4034delA founder mutations (OR = 2.98, 95%CI = 1.58 to 5.62, P < 0.001). We have also found a difference in the prevalence of breast and ovarian cancer cases among the 1^st ^and 2^nd ^degree relatives of the c.4034delA and c.5266dupC mutation carriers. In addition, among the breast cancer cases the c.4034delA mutation has been associated with a later age of onset and worse clinical outcomes in comparison with the c.5266dupC mutation.

**Conclusions:**

Our data suggest that the carriers of the c.4034delA and c.5266dupC founder mutations have different risks of breast and ovarian cancer development, different age of onset and prognosis of breast cancer.

## Background

Breast cancer is the most prevalent malignancy and the leading cause of death from cancer among women in Latvia. Ovarian cancer appears less frequent; however, it remains a significant cause of cancer mortality in Latvia and worldwide. Hereditary cancer syndromes account for up to 5-10% of breast cancer cases and for up to 5-15% of ovarian cancer cases [1,2]. Germline mutations of the *BRCA1 *and *BRCA2 *genes represent the most significant and thus far the best characterized genetic risk factors for breast and ovarian cancer development [3]. More than 1000 distinct cancer-associated *BRCA1 *[MIM 113705] mutations have been already described; however, not all of them are equally pathogenic and most probably there is a different cancer risk associated with specific mutations [4]. In sporadic breast carcinoma, *BRCA1 *is rarely mutated, although expression frequently is limited by DNA methylation-induced gene suppression. The *BRCA1 *protein has been implicated in different biological processes, including DNA repair, cell cycle control, transcriptional regulation, centrosome duplication and tumour suppressor function [5].

Structural and functional changes of mutated proteins caused by different *BRCA1 *mutations are not identical and can lead to various phenotypes of cancers (genotype-phenotype correlations) [6]. Therefore, clinical presentations, outcome and response to treatment of tumours can differ significantly depending on the type of mutations. In the case of pathogenic *BRCA1 *mutations, it has been previously shown that mutations in exon 11 (nucleotides 2388-4185) of the *BRCA1 *gene are associated with almost equal breast and ovarian cancer incidence among mutation carriers (breast:ovarian cancer ratio) in comparison with mutations in other parts of the *BRCA1 *gene. On the other hand, mutations located 3' of nucleotide 4185 are usually associated with a higher risk of breast cancer development and with a relatively lower ovarian cancer risk [2,6]. Nevertheless, the genotype-phenotype correlations of the specific *BRCA1 *founder mutations c.4034delA (also described as 4153delA (or 4154delA) in exon 11) and c.5266dupC (5382insC in exon 20) have not been fully investigated. In this article, we describe some aspects of genotype-phenotype correlation among the c.4034delA and c.5266dupC mutation carriers identified in a population-based screening in Latvia which was observed in the prevalence of breast and ovarian cancer cases among the mutation carriers and their 1^st ^and 2^nd ^degree relatives, in the age of onset and in the clinical outcomes of breast cancer.

## Methods

The study population is comprised of 2546 unselected breast cancer patients and 795 unselected ovarian cancer patients who, during the period from 2000 to 2009, underwent genetic counselling and *BRCA1 *founder mutation analysis at the Hereditary Cancer Outpatient Clinic of Pauls Stradins Clinical University Hospital in Riga, Latvia. Screening for the *BRCA1 *founder mutations c.4034delA and c.5266dupC was performed in different patient groups as follows: 1) in all the breast and ovarian cancer patients irrespective of family history (there were no exclusion criteria); 2) in patients with other cancer localizations and in healthy individuals when hereditary breast and ovarian cancer syndromes were suspected. The study was approved by the Ethical Committee of Riga Stradins University. All participants signed informed consent forms for participation in this study.

The information about the time of establishing breast cancer diagnosis, the TNM classification and the time and cause of death of cancer patients was confirmed in the Latvian Ministry of Health, the "Register of Patients Suffering from Particular Diseases, Patients with Cancer". The patients for the control group for breast cancer survival analysis were randomly selected from the breast cancer patients who were tested negative for the *BRCA1 *founder mutations, matched by tumour size, nodal status and age at diagnosis. Oestrogen receptor (ER), progesterone receptor (PR) and HER2/neu expression status was assessed using a standard immunohistochemical technique. The data on family histories were obtained from the database of the Hereditary Cancer Institute (in Riga Stradins University) as reported by probands. During genetic counselling the following data were collected using special questionnaires: the number of cancer cases among the 1^st ^- 4^th ^degree relatives in the family with indication of cancer localization in the affected relatives, the age of onset of cancer disease and the age of death. The presence of *BRCA1 *founder mutations c.4034delA and c.5266dupC was determined as described elsewhere [7].

Statistical analysis was performed using EpiCalc 2000 version 1.02 and SPSS, version 19. We estimated the survival function with the Kaplan-Meier estimator and calculated cumulative incidences as one minus survival function. Differences in survival and cumulative incidence were compared with the Log Rank test. Adjusted Hazard Ratios (HR) were estimated with Cox proportional hazard regression. Association between cancer type and mutation was assessed with Odds Ratios (OR) and associated Mantel-Haenszel 95% Confidence Intervals (CI). Comparison of two independent proportions was performed by Z-test.

## Results

### 1. Different breast:ovarian cancer ratios among c.4034delA and c.5266dupC mutation carriers

Molecular analysis of *BRCA1 *founder mutations among 2546 unselected breast cancer patients has revealed the presence of mutations in 96 (3.77%) cases, including 25 (0.98%) c.4034delA mutations and 69 (2.70%) c.5266dupC mutations. From 795 unselected ovarian cancer patients *BRCA1 *mutations were identified in 79 (9.90%) cases, including 41 (5.10%) c.4034delA and 38 (4.80%) c.5266dupC mutations.

Among the 107 breast and ovarian cancer patients who were identified as the mutation c.5266dupC carriers, breast cancer cases accounted for 64%; however, among the 76 breast and ovarian cancer patients c.4034delA mutation carriers breast cancer cases accounted only for 32% (OR = 2.98, 95% CI = 1.58 to 5.62, P < 0.001), which reflected the difference in the breast:ovarian cancer ratios among the carriers of these founder mutations. The difference in the breast:ovarian cancer relative risk associated with the c.5266dupC and c.4034delA founder mutations in this population-based series was calculated as RR = 1.70 (95% CI = 1.21 to 2.39, P < 0.001).

### 2. Age at diagnosis of breast and ovarian cancer

**Figure 1:**
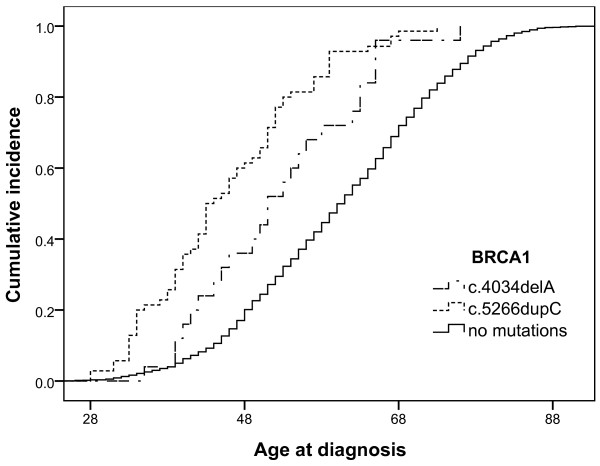
**Age-related cumulative incidence of breast cancer cases among c.4034delA and c.5266dupC mutation carriers and patients without mutations**.

In this population-based series the median age at diagnosis of breast cancer patients among the cases with no mutations was 60.21 (age range 21-95) years in comparison with 51.76 (age range 35-76) years among the c.4034delA mutation carriers and 46.51 (age range 28-76) years among the c.5266dupC mutation carriers. The median age at diagnosis of ovarian cancer cases among the cases without mutations was 58.57 (age range 30-88) years in comparison with 53.21 (age range 28-73) years among the c.4034delA mutation carriers and 49.30 (age range 35-77) years among the c.5266dupC mutation carriers. The linear trends of age-related cumulative incidence of breast cancer cases are shown in Figure 1. We observed a significant difference in cumulative incidence of breast cancer among the c.5266dupC and c.4034delA mutation carriers (χ^2 ^= 4.39, with 1 degree of freedom, P = 0.03). The difference in cumulative incidence of patients without mutations in comparison with the c.5266dupC (χ^2 ^= 137.57, with 1 degree of freedom, P < 0.001) and c.4034delA (χ^2 ^= 15.27, with 1 degree of freedom, P = P < 0.001) mutation carriers was also statistically significant. The difference in age at diagnosis of ovarian cancer among the c.4034delA and c.5266dupC mutation carriers and patients without mutations was not statistically significant (data not shown).

### 3. Survival analysis of breast cancer patients

The breast cancer survival analysis includes 25 mutation c.4034delA carriers, 68 mutation c.5266dupC carriers and 103 patients without mutations, with a median follow-up period of 103 months. There were 36 women who died from breast cancer (7 in the c.4034delA group, 11 in the c.5266dupC group and 18 in the control group) and 13 women who died from causes other than breast cancer: 5 women died from ovarian cancer (1 in the c.4034delA group, 3 in the c.5266dupC group and 1 in the control group), 4 from cancers of other sites (1 in the c.4034delA group, 1 in the c.5266dupC group and 2 in the control group) and 4 deaths unrelated to cancer. The last 4 cases were included in survival analysis, but were counted as the end of the follow-up period and not as death events.

**Figure 2:**
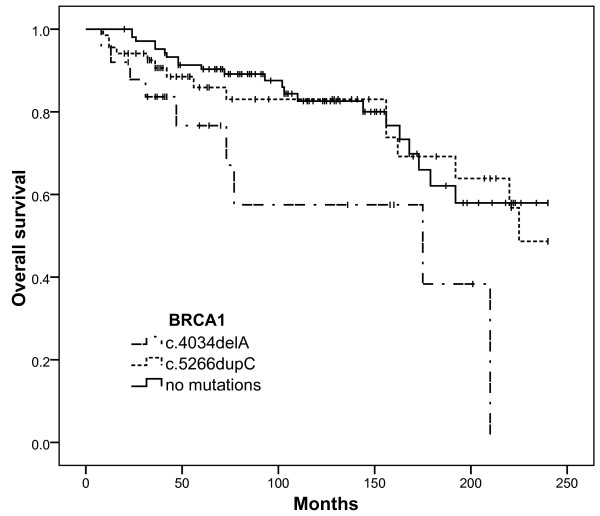
**Overall survival of breast cancer patients - c.4034delA and c.5266dupC mutation carriers and patients without mutations**.

The mean estimated survival time during a 20 year follow-up period was calculated as 134 months (95% CI = 92.58 to 177.00) in the c.4034delA group in comparison with 189 months in the c.5266dupC group (95% CI = 166.75 to 211.43) and 192 months in the control group (95% CI = 175.54 to 208.83). The cumulative survival plot is shown in Figure 2. Analysis of the Kaplan-Meier curves showed that the clinical outcome of breast cancer patients who were the c.4034delA mutation carriers was significantly worse in comparison with the c.5266dupC mutation carriers (χ^2 ^= 4.32, with 1 degree of freedom, P = 0.038) and with patients from the control group (χ^2 ^= 7.05, with 1 degree of freedom, P = 0.008). We did not observe any significant difference in tumour staging and lymph node status in both groups of mutation carriers (Table 1).

**Table 1:** Clinical characteristics of patients included in the survival analysis

Variable	c.4034delA	c.5266dupC	Control
N. of patients	25	68	103
Median age of onset (years)	51.7	46.5	54.9
T4 - n. (%)	5 (20%)	10 (15%)	7 (7%)
T3 - n. (%)	1 (4%)	3 (4%)	13 (13%)
T2 - n. (%)	14 (56%)	43 (63%)	63 (61%)
T1 - n. (%)	5 (20%)	12 (18%)	20 (19%)
Axillary node positive - n. (%)	14 (56%)	32 (47%)	44 (43%)
ER/PR positive (%)	50%	15%	70%
HER2 positive (%)	22%	11%	10%
ER/PR/HER2 (Triple) negative (%)	50%	79%	24%
N. of patients dead from cancer during follow-up period	9 (36%)	15 (22%)	21 (20%)

We also performed Cox regression analysis among breast cancer patients where cancer related mortality was used as the end point. The presence of the c.4034delA founder mutation remained an independent predictor of unfavourable prognosis in multivariable analysis among *BRCA1 *mutation carriers (Table 2). The presence of any *BRCA1 *founder mutation was not significantly associated with unfavourable prognosis in multivariable analysis among all hereditary and sporadic breast cancer patients (Table 3). Hormone receptor status was not included in multivariate analysis, because it was available only for 52% of the patients. However, in univariable analysis hormone receptor negativity was significantly associated with unfavourable prognosis only among the patients without *BRCA1 *mutations (HR = 0.18, 95% CI = 0.03 to 0.98, P = 0.04), whereas the impact of hormone receptor status on the prognosis of breast cancer among *BRCA1 *mutation carriers was not statistically significant (HR = 1.09, 95% CI = 0.21 to 5.67, P = 0.91).

**Table 2:** Multivariable Cox-regression analysis of *BRCA1 *c.4034delA and c.5266dupC mutation carriers breast cancer patients

Variable	n	HR	95%CI	P-value
*BRCA1 *mutations:				
c.4034delA	25	2.76	1.13 - 6.70	0.02
c.5266dupC	68			
Tumor size:				
< 5 cm	74	0.36	0.15 - 0.86	0.02
> 5 cm	19			
Axillary node:				
Negative	44	0.34	0.13 - 0.89	0.03
Positive	49			
Age at diagnosis:				
< 50	51	1.53	0.65 - 3.61	0.32
> 50	42			

**Table 3:** Multivariable Cox-regression analysis of breast cancer patients

Variable	n	HR	95%CI	P-value
*BRCA1 *mutations:				
Mutation present	93	1.10	0.81 - 1.48	0.54
Mutation absent	103			
Tumor size:				
< 5 cm	157	0.68	0.49 - 0.94	0.02
> 5 cm	39			
Axillary node:				
Negative	103	0.34	0.37 - 0.77	< 0.01
Positive	93			
Age at diagnosis:				
< 50	89	1.19	0.66 - 2.17	0.55
> 50	107			

### 4. Different prevalence of breast and ovarian cancer cases among relatives of c.4034delA and c.5266dupC mutation carriers

Hereditary cancer institute database contains information about 207 families of *BRCA1 *founder mutation carriers (including 79 families of c.4034delA and 128 families of c.5266dupC mutation carriers) who were identified during genetic screening in Latvia. Overall, the c.4034delA mutation carriers reported about cancer cases in their families more frequently in comparison with the c.5266dupC mutation carriers (the average amount of individuals ill with cancer in c.4034delA families was 2.9 individuals per family in comparison with 2.1 individuals per family reported in the c.5266dupC group, P = 0.009). The amount of "breast cancer families" with a history of only breast cancer cases and without ovarian cancer cases (at least two 1^st ^or 2^nd ^(related through a man) degree relatives) was higher among the c.5266dupC mutation carriers, whereas the amount of "breast and ovarian cancer families" with a history of ovarian cancer cases with or without breast cancer cases was higher among the carriers of the c.4034delA mutation. Probands, c.4034delA mutation carriers, have reported more frequently about multiple cases of any cancer (3 or 4) among the 1^st ^and 2^nd ^degree relatives in their families in comparison with the c.5266dupC mutation carriers (Table 4).

**Table 4:** Family histories of *BRCA1 *c.4034delA and c.5266dupC mutations carriers

Types of family histories	c.4034delA	c.5266dupC	95% CI	P-value
No data about cancer cases among 1^st ^and 2^nd ^degree relatives	9% (7)	22%(28)	2.44-23.56	0.02
At least 3 cancer cases among 1^st ^and 2^nd ^degree relatives (including breast and ovarian)	48% (38)	29% (37)	4.46-33.54	0.008
At least 3 cancer cases among 1^st ^and 2^nd ^degree relatives (without breast and ovarian)	14% (11)	6% (8)	-2.05-17.45	0.08
At least 4 cancer cases among 1^st ^and 2^nd ^degree relatives (including breast and ovarian)	30% (24)	15% (19)	2.14-27.86	0.01
Breast cancer families^1^	9% (7)	24% (31)	4.25-25.75	0.01
Breast and ovarian cancer families^2^	34% (27)	18% (23)	2.61-29.39	0.01
Total amount of families included	79	128		

^1 ^- Breast cancer families - all families with at least two 1^st ^or 2^nd ^(related through a man) degree relatives having breast cancer but without ovarian cancer cases in family.

^2 ^- Breast and ovarian cancer families - all families with at least two 1^st ^or 2^nd ^(related through a man) degree relatives having breast or ovarian cancers.

Due to the polygenic inheritance of breast and ovarian cancers, clustering of specific cancer localizations within families can be considered an important factor for unspecific bias of results obtained by analysing family histories. To overcome this problem we also investigated the prevalence of breast, ovarian and other cancer localizations among all the 1^st ^and 2^nd ^degree relatives of the carriers of both *BRCA1 *founder mutations irrespective of family composition. The prevalence of different cancer localizations among all the 1^st ^and 2^nd ^degree relatives of the c.4034delA and c.5266dupC mutation carriers is shown in Table 5.

**Table 5:** Prevalence of different cancer localizations among 1^st ^and 2^nd ^degree relatives of probands who were *BRCA1 *c.4034delA and c.5266dupC mutation carriers (in % of all  the reported cancer cases in each group)

Cancer site	c.4034delA	c.5266dupC	95% CI	P-value
Breast	19.0%	34.0%	7.16 to 22.84	0.0002
Ovary	19.0%	8.0%	4.66 to 17.34	0. 0003
Other gynaecological cancers*	16.0%	8.0%	1.94 to 14.06	0.006
Colon and rectum	2.6%	3.1%	-2.75 to 3.75	n.s
Pancreas	1.3%	1.3%	-2.35 to 2.35	n.s
Prostate	1.7%	2.4%	-2.12 to 3.52	n.s
Stomach	11.3%	9.6%	-4.00 to 7.40	n.s
Lung	6.5%	2.7%	-0.28 to 7.88	0.059
Total amount of relatives reported as having cancer	230	290		

* - Other gynaecological cancers include ovarian, uterine and cervical cancer cases when exact localization of cancer was unknown.

n.s. - non-significant

## Discussion

In this study we investigated the prevalence of the most common *BRCA1 *founder mutations in a population-based series of breast and ovarian cancer cases in Latvia. Over the past several years, genetic counselling and screening of *BRCA1 *founder mutations have covered more than 50% of all breast and ovarian cancer cases registered in Latvia each year, thus providing information about the prevalence of hereditary cancer syndromes and *BRCA1 *founder mutations in unselected groups of breast and ovarian cancer patients. The fact that we investigated the prevalence of only 2 specific founder mutations is the major limitation of our study; however, according to the already published data, these 2 founder mutations account for more than 80% of all the *BRCA1 *mutations that were found among the cancer patients in Latvia [8,9]. Several population-based studies performed in neighbour countries of Latvia (Lithuania, Belarus and Poland) have demonstrated that 3 founder mutations - c.4034delA, c.5266dupC and c.181T > G (300T/G) - are the most widespread *BRCA1 *founder mutations in the southern Baltic region [10-14]. Previous studies in Latvia have shown that the c.181T/G mutation accounts for only 6-10% of the identified *BRCA1 *mutations when genetic analysis had been performed in selected early-onset breast and ovarian cancers patients [8] or in specific Latvian regions [9]. Nevertheless, according to our estimation the prevalence of the c.181T/G founder mutation among unselected breast and ovarian cancer patients in Latvia is much lower. The exact prevalence of *BRCA1 *founder mutations in Latvia is difficult to investigate due to relatively high heterogeneity of the Latvian population; however, in the analysis of a population screening of hereditary cancer syndromes in the Valka district of Latvia, the prevalence of *BRCA1 *founder mutations in the Latvian population was estimated at approximately 0.05% [7].

In our study we also found some phenotypic variations of hereditary breast and ovarian cancer syndromes among the c.4034delA and c.5266dupC mutation carriers. First of all, we observed a significant difference in the prevalence of c.4034delA and c.5266dupC mutation carriers among the breast and ovarian cancer patients. The breast:ovarian cancer ratio was higher among the c.5266dupC mutation carriers and lower among the c.4034delA mutation carriers. Despite the fact that some previous reports have shown an almost equal breast:ovarian cancer ratio among the c.4034delA and c.5266dupC mutation carriers in Latvia, in these studies genetic analysis was performed in relatively small hospital-based cohorts that can explain these discrepancies [8,9]. *BRCA1 *c.4034delA was initially described as a low penetrance breast cancer mutation [15]. Several other reports have demonstrated an increased prevalence of ovarian cancer cases among the c.4034delA mutation carriers [12-14]. In the analysis of hospital-based series of breast and ovarian cancer cases in Belarus the breast:ovarian cancer ratio among the c.4034delA and c.5266dupC mutation carriers was similar to the breast:ovarian cancer ratio obtained in our population-based series [13].

Several studies have shown some other phenotypic variations besides breast:ovarian cancer ratios associated with mutations, located in different parts of the *BRCA1 *gene. Satagopan et al found that the estimated lifetime risk of ovarian cancer development were two times as high for the c.68_69delAG (185delAG) mutation (66%) than for the c.5266dupC mutation (29%) [16]. Al-Mulla et al showed that age-related expressivity and penetrance of breast and ovarian cancers depended on the mutation position in the *BRCA1 *gene and differed among the carriers of various mutations located in exons 2, 11 and 13 [17].

The genotype-phenotype correlation effect among the c.4034delA and c.5266dupC mutation carriers observed in our study had two other interesting features. The median age of onset of breast and ovarian cancers among the c.5266dupC mutation carriers was smaller than among the c.4034delA mutation carriers. A similar trend in the age of onset of breast cancer cases among the c.4034delA and c.5266dupC mutation carriers was previously shown in a hospital-based series of Belarusian breast cancer patients; however, in this report the median age at diagnosis of breast cancer was lower than in our population-based series (48 years in patients without mutations, 43 years in c.5266dupC carriers and 44 years in c.4034delA carriers) [13].

Despite many conflicting reports about the prognostic significance of *BRCA1 *mutations in breast cancer patients (where patients with different mutations were usually combined in common groups) [18-20], the influence of individual *BRCA1 *mutations on the clinical outcomes of breast cancer patients has not been fully investigated. In our study we observed a worse clinical outcome for the c.4034delA mutation carriers in comparison with the c.5266dupC mutation carriers and the sporadic breast cancer patients. To some extent, this observation can be attributed to the way a part of the patients were referred to genetic counselling. In many cases, patients were counselled when they were admitted to hospital due to a recurrence of the cancer process or appearance of cancers in other sites. Among the c.5266dupC mutation carriers, more than 20% of breast cancer patients were diagnosed with contralateral breast cancer, when the primary site had been successfully treated in some cases more than 20 years ago. Among the carriers of the c.4034delA mutation such cases were observed less frequently (there was only one case with a disease-free period of 10 years until contralateral breast cancer was diagnosed); however, there was a substantial amount of patients with a progression of primary breast cancer processes which dramatically reduced survival in many cases. In spite of this fact, worse overall survival among the c.4034delA mutation carriers was not associated with an advanced stage at diagnosis. The amount of cases with locally advanced and node positives breast cancers did not differ significantly among all groups of patients included in the survival analysis. Furthermore, the multivariable analysis has shown that the presence of the c.4034delA mutation remained an independent predictive factor. On the other hand, improved survival among the c.5266dupC mutation carriers could not be attributed to prophylactic surgery or increased surveillance strategies. Indeed, these options have become available for the breast cancer patients with *BRCA1 *mutations in Latvia just recently which might explain a relatively high amount of contralateral breast cancer cases among the carriers of both *BRCA1 *founder mutations.

Taken together, all the described above features show several aspects of genotype-phenotype correlation among the carriers of 2 specific *BRCA1 *founder mutations. Based on these data we can suggest that the carriers of the c.4034delA and c.5266dupC founder mutations have different risks of breast and ovarian cancer development, different age of onset and prognosis of breast cancer. The evaluation of the exact risk of specific genetic alterations in breast and ovarian susceptibility genes and possible synergistic effects between them can lead to a more precise prediction of the individual risk of developing specific cancers in hereditary breast and ovarian cancer patients. According to the suggestions of Mavaddat et al further decision about cancer surveillance and risk reducing strategies should take into account the individual risk differences based on the evaluation of the status of different genetic susceptibility factors [3]. For example, due to the genotype-phenotype correlation effect, carriers of the c.4034delA mutation have a more significant predisposition to ovarian cancer than the c.5266dupC mutation carriers. This can suggest a much stronger indication of risk-reducing salpingo-oophorectomy among the carriers of this founder mutation. Although the c.5266dupC founder mutation in this study was associated with better prognosis, the risk of breast cancer among asymptomatic mutation carriers as well as the risk of contralateral breast cancer among breast cancer patients, carriers of this founder mutation, remains high. This indicates the necessity of intensive surveillance strategies with a recommendation of risk-reducing bilateral mastectomy for the carriers of this mutation. Several studies have shown that risk-reducing bilateral mastectomy reduces the risk of breast cancer among BRCA1 mutation carriers by approximately 90% and additional risk-reducing bilateral salpingo-oophorectomy improves this parameter by only 5% to a total of 95% [21,22]. These data suggest that for carriers of the c.5266dupC mutation risk-reducing bilateral mastectomy without salpingo-oophorectomy can be sufficient risk-reducing option, especially for women of childbearing age.

Despite their approved effectiveness, most cancer preventive strategies frequently reduce the quality of life of patients and are usually associated with a unique set of social, emotional and sexual factors for *BRCA1 *mutation carriers and their family members which can influence the decision making processes in many cases [23,24]. This issue indicates the importance of further clinical studies to evaluate the significance of different cancer prevention options among the carriers of specific genetic alterations in *BRCA1 *and other breast and ovarian cancer susceptibility genes.

## Conclusions

In a population-based series of unselected breast and ovarian cancer cases in Latvia, the prevalence of breast cancer cases among the c.5266dupC mutation carriers was significantly higher in comparison with the c.4034delA mutation carriers, whereas the prevalence of ovarian cancer cases was almost similar. Among the c.4034delA mutation carriers, breast cancers were diagnosed at a later age and had worse clinical outcomes in comparison with the c.5266dupC mutation carriers. Based on our data we can suggest that carriers of the c.4034delA and c.5266dupC founder mutations have different risks of breast and ovarian cancer development, different age of onset and prognosis of breast cancer.

## Competing interests

The authors declare that they have no competing interests.

## Authors' contributions

GP conceived, designed and coordinated the study, performed statistical analysis and drafted the manuscript. AI participated in the analysis of data, coordinated the study and helped to draft the manuscript. AG, SS, SR, MB, GK participated in the analysis of data and provided study material. GP, GT helped to draft the manuscript. UT supervised the statistical analysis. EM performed the molecular examinations, participated in the analysis of data and helped to draft the manuscript. JG coordinated the study and helped to draft the manuscript. All authors read and approved the final manuscript.

## Pre-publication history

The pre-publication history for this paper can be accessed here:

http://www.biomedcentral.com/1471-2350/12/147/prepub
